# Suspected Macrolide‐Refractory *Mycoplasma pneumoniae* Pneumonia With Discordant *Coccidioides* Serology in an Immunocompromised Host: A Case Report

**DOI:** 10.1155/crdi/7967464

**Published:** 2026-06-26

**Authors:** Mohamed M. G. Mohamed, Garry Francis Morel, Matthew Stack, John Salomone, Ariel Rischall, Ghassan Kamel

**Affiliations:** ^1^ Division of Pulmonary, Critical Care, & Sleep Medicine, Internal Medicine Department, Saint Louis University School of Medicine, Saint Louis, Missouri, USA, slu.edu; ^2^ Division of Infectious Diseases, Allergy and Immunology, Internal Medicine Department, Saint Louis University School of Medicine, Saint Louis, Missouri, USA, slu.edu; ^3^ Division of Hematology and Medical Oncology, Saint Louis University School of Medicine, Saint Louis, Missouri, USA, slu.edu

**Keywords:** case report, coccidioidomycosis, false-positive serology, hypogammaglobulinemia, immunocompromised host, *Mycoplasma pneumoniae*, suspected macrolide-refractory disease

## Abstract

Suspected macrolide‐refractory *Mycoplasma pneumoniae* pneumonia can be a diagnostic challenge in immunocompromised hosts, particularly after allogeneic stem cell transplantation. Screening serologies may further complicate diagnosis when false‐positive or discordant results divert therapy. We report a 63‐year‐old woman with B‐cell acute lymphoblastic leukemia status postallogeneic stem cell transplantation and hypogammaglobulinemia who was transferred for persistent cough, exertional dyspnea, and right upper‐lobe consolidation after limited improvement with azithromycin and broad‐spectrum antibacterial therapy. Initial and repeat respiratory testing detected *M. pneumoniae*, while an extensive infectious evaluation was otherwise negative. *Coccidioides* enzyme immunoassay IgM and IgG were positive, prompting empiric fluconazole, but confirmatory immunodiffusion and complement fixation testing were negative, and the patient lacked epidemiologic risk factors. Following reassessment, fluconazole was discontinued; doxycycline and replacement intravenous immunoglobulin were initiated. Clinical improvement followed, including discontinuation of supplemental oxygen and return to baseline exercise tolerance, although the relative contributions of doxycycline and intravenous immunoglobulin cannot be determined from a single case. This case highlights the need to interpret screening fungal serologies in a clinical and epidemiologic context, confirm positive *Coccidioides* EIA results, and consider clinically suspected macrolide‐refractory *M. pneumoniae* when respiratory symptoms persist despite macrolide therapy. Molecular resistance testing was not available, so macrolide resistance remained clinically suspected rather than proven.

## 1. Introduction

*Mycoplasma pneumoniae* (*M. pneumoniae*) is an atypical bacterium that causes respiratory infections ranging from upper respiratory tract illness to community‐acquired pneumonia. Although *M. pneumoniae* infection is not nationally notifiable in the United States, CDC surveillance notes that infections are common, may occur at any time of year, can be more common in summer and early fall, and usually peak every 3–7 years [1]. Pneumonia remains one of the most clinically important manifestations [2]. In a large US study of adults hospitalized with community‐acquired pneumonia, approximately 2% tested positive for *M. pneumoniae* [3].

Macrolides are commonly used first‐line agents for *M. pneumoniae* infections; however, confirmed macrolide resistance and persistent disease despite macrolide therapy are increasingly recognized clinical concerns [4]. CDC data suggest that macrolide resistance remains less than 10% overall in the United States, although higher proportions have been reported in selected regions, clusters, and outbreaks [1]. Regional US data, including Midwestern surveillance, also demonstrate that local resistance prevalence may vary [5]. In immunocompromised hosts, diagnosis can be further complicated by prolonged symptoms, atypical radiographic findings, limited immune responses, and nonspecific or false‐positive serologic tests.

We report a diagnostically challenging case of suspected macrolide‐refractory *M. pneumoniae* pneumonia in an immunocompromised patient with discordant *Coccidioides* serology. Because cold‐agglutinin testing and molecular macrolide‐resistance testing were not performed, the mechanism of the discordant serology and the resistance phenotype are interpreted cautiously rather than presented as proven findings.

## 2. Case Presentation

A 63‐year‐old woman with a history of B‐cell acute lymphoblastic leukemia treated with allogeneic peripheral stem cell transplantation in 2015 and hypogammaglobulinemia after a 7‐month trial off intravenous immunoglobulin (IVIG) was transferred to our facility for evaluation of persistent respiratory symptoms. She initially presented to an outside facility with several days of significant dry cough and exertional dyspnea. She also reported low‐grade fever, generalized malaise, and fatigue, but denied chest pain, chest tightness, or wheezing. Chest imaging demonstrated right upper‐lobe consolidation, and *M. pneumoniae* was detected by respiratory molecular testing. She was treated for community‐acquired pneumonia, but her clinical response remained limited despite azithromycin, ceftriaxone, and piperacillin–tazobactam, as evidenced by persistent cough, exertional dyspnea, ongoing oxygen need, and persistent radiographic abnormality; this prompted transfer to our facility.

On arrival at our center, she was ill appearing and hypoxemic, requiring 2 L/min of supplemental oxygen. Vital signs were otherwise stable. Examination showed no lymphadenopathy or cyanosis, and chest and abdominal examinations were otherwise unremarkable.

Repeat respiratory molecular testing confirmed *M. pneumoniae*, and chest computed tomography showed persistent right upper‐lobe consolidation, scattered ground‐glass opacities, and tree‐in‐bud nodules (Figure 1). Extensive microbiological evaluation, including bacterial and viral panels; *Legionella* and *Streptococcus pneumoniae* urinary antigens; and fungal assays for *Histoplasma*, *Blastomyces*, *Cryptococcus*, and beta‐D‐glucan, was negative. Serological testing revealed positive *Coccidioides* enzyme immunoassay IgM and IgG, raising concern for possible coccidioidomycosis (Table 1). However, because the patient lacked epidemiologic risk factors, false‐positive screening results were suspected, and confirmatory immunodiffusion and complement fixation testing were ordered. Empiric fluconazole 400 mg daily was initiated while confirmatory testing was pending.

**FIGURE 1:**
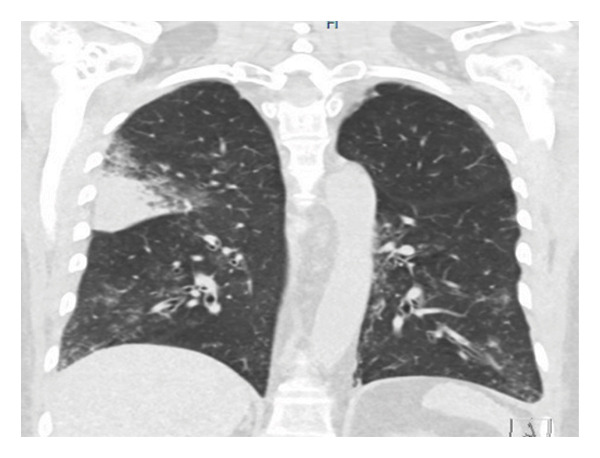
Chest computed tomography without contrast showing right upper‐lobe consolidation, scattered ground‐glass opacities, and tree‐in‐bud nodules.

**TABLE 1:** Infectious workup laboratory results.

Test	Specimen	Result
*Bordetella pertussis* PCR	Nasopharyngeal	Not detected
*Bordetella parapertussis* PCR	Nasopharyngeal	Not detected
*Chlamydia pneumoniae* PCR	Nasopharyngeal	Not detected
*Mycoplasma pneumoniae* PCR	Nasopharyngeal	Detected
MRSA DNA by PCR	Nasopharyngeal	Not detected
*Legionella* antigen	Urine	Negative
*Streptococcus pneumoniae* antigen	Urine	Negative
*Histoplasma* antibody	Blood	Not detected
Histoplasma yeast CF antibody	Blood	< 1:8 (negative)
*Blastomyces* antibody (ID)	Blood	Not detected
*Coccidioides* antibody IgG	Blood	1.0 (high)
*Coccidioides* antibody IgM	Blood	2.8 (high)
*Cryptococcus* antigen	Blood	Negative
Beta‐d‐glucan	Blood	< 31 (negative)
*Aspergillus* DNA PCR	Blood	Negative
Pneumococcal serotype 1 antibody IgG	Blood	Negative
Tetanus antibody	Blood	0.1 (negative)

Abbreviations: CF, complement fixation; DNA, deoxyribonucleic acid; IgG/IgM, immunoglobulin G/immunoglobulin M; MRSA, methicillin‐resistant *Staphylococcus aureus*; PCR, Polymerase chain reaction; RT‐PCR, reverse transcription polymerase chain reaction.

The patient underwent bronchoscopy with bronchoalveolar lavage, which showed a neutrophil‐predominant lavage. Repeat PCR testing detected only *M. pneumoniae*. The patient stabilized clinically and was discharged on fluconazole with outpatient follow‐up while confirmatory fungal testing was pending. The *Coccidioides* immunodiffusion and complement fixation tests later returned negative. Within 7 days of discharge, she was readmitted with nausea, vomiting, and diarrhea complicated by hyponatremia. These gastrointestinal symptoms were new relative to the initial respiratory presentation. Given the negative fungal confirmatory tests and persistent or recurrent illness after macrolide exposure, a suspected macrolide‐refractory *M. pneumoniae* infection was considered. Fluconazole was discontinued, doxycycline 100 mg twice daily for 7 days was initiated, and IVIG was restarted at replacement dosing. Following doxycycline and IVIG, her respiratory and gastrointestinal symptoms improved substantially. She no longer required supplemental oxygen, regained baseline exercise tolerance, and was discharged home in good condition with satisfactory recovery.

## 3. Discussion

This case illustrates the diagnostic difficulty of persistent atypical pneumonia in an immunocompromised host. The patient had repeated detection of *M. pneumoniae* and limited response after macrolide exposure, but the diagnostic pathway was diverted by positive screening *Coccidioides* EIA IgM and IgG results. Confirmatory immunodiffusion and complement fixation testing were negative, and she lacked epidemiologic risk factors for coccidioidomycosis; these findings made fungal pneumonia less likely and supported reconsideration of *M. pneumoniae* as the principal pathogen. The case is therefore best framed as a diagnostic challenge with clinically suspected, rather than proven, macrolide‐refractory disease.

Serologic tests are useful but imperfect, especially when interpreted without exposure history, pretest probability, and confirmatory testing [6]. Cross‐reactivity and discordant serologic results have been described with atypical bacterial infections, including *M. pneumoniae* [7]. The literature on false‐positive *Coccidioides* EIA results is mixed, but several studies emphasize that EIA specificity varies by clinical context. Kuberski et al. reported that 82% of patients with isolated IgM‐positive/IgG‐negative EIA results did not have coccidioidomycosis, highlighting the risk of overdiagnosis from screening serology alone [11]. Blair et al. similarly found that the clinical specificity of *Coccidioides* EIA depends on why testing is performed [9]. Donovan et al. emphasize that interpretation of coccidioidomycosis serology remains a common diagnostic challenge and should be integrated with clinical syndrome, epidemiology, and confirmatory testing [12].

In our patient, a false‐positive *Coccidioides* EIA result is biologically plausible but not proven. *M. pneumoniae* infection can trigger IgM‐mediated immune phenomena such as cold agglutinins [8], and nonspecific IgM immune activation may contribute to misleading screening serologies. However, cold‐agglutinin testing was not performed, and no direct assay demonstrated cross‐reactivity between this patient’s *M. pneumoniae* infection and the *Coccidioides* EIA. Therefore, this mechanism remains speculative and should not be interpreted as established in this case.

Best practice in this scenario is to confirm positive *Coccidioides* EIA results with immunodiffusion and complement fixation, especially when exposure risk is low or the clinical syndrome is atypical. CDC guidance recommends EIA with immunodiffusion or complement fixation for initial evaluation and states that follow‐up ID/CF testing can help rule out false positives after a positive EIA; if EIA is positive but ID/CF is negative and clinical suspicion remains, repeat ID/CF testing in 2–4 weeks can be considered [13]. In immunosuppressed patients or rapidly progressive illness, microscopy and culture from bronchoscopy specimens, PCR, antigen testing, and specialist consultation may also be considered [13].

The terminology of macrolide resistance also requires caution. Proven macrolide‐resistant *M. pneumoniae* generally requires molecular evidence, such as detection of 23S rRNA mutations, or phenotypic susceptibility testing [4]. In this case, available records did not document molecular resistance testing or susceptibility testing. Thus, the case is best described as suspected macrolide‐refractory *M. pneumoniae* pneumonia or clinically suspected macrolide resistance rather than proven macrolide‐resistant infection. The suspicion was supported by persistent symptoms and oxygen requirement after macrolide therapy, recurrence/readmission, negative alternative microbiological evaluation, and subsequent improvement after doxycycline and IVIG; however, these observations do not confirm a resistance mechanism.

Restarting IVIG may also have contributed to recovery because the patient had hypogammaglobulinemia and had been off IVIG for several months. Adjunctive IVIG has been studied in refractory *M. pneumoniae* pneumonia, although available evidence is limited and much of it comes from pediatric populations [10]. Because doxycycline and IVIG were initiated together, the relative contribution of each intervention cannot be determined from this single case. The observed improvement should therefore be interpreted as a temporal association rather than proof of causality or therapeutic superiority.

## 4. Conclusion

In immunocompromised patients with persistent atypical pneumonia after macrolide therapy, clinicians should consider ongoing *M. pneumoniae* infection with clinically suspected macrolide‐refractory disease while seeking confirmatory resistance testing when available. Positive *Coccidioides* EIA results should be interpreted in light of epidemiologic risk and confirmed with immunodiffusion, complement fixation, or additional microbiologic methods to avoid misdiagnosis and delayed appropriate therapy. Improvement after doxycycline and IVIG in this case should be interpreted cautiously because both were initiated together and their individual contributions are uncertain.

## Funding

This research received no specific grant from any funding agency in the public, commercial, or not‐for‐profit sectors.

## Disclosure

This manuscript has not been posted as a preprint and has not been presented at a scientific conference or seminar.

## Ethics Statement

Institutional review board approval was not required for this single‐patient case report according to institutional policy.

## Consent

Written informed consent for publication of this case report and any accompanying images was obtained from the patient.

## Conflicts of Interest

The authors declare no conflicts of interest.

## Data Availability

All de‐identified data relevant to this case are included within the manuscript and the separately submitted table and figure files. Additional de‐identified clinical details may be available from the corresponding author upon reasonable request and subject to institutional requirements.
